# Inhibition of 7α,26-dihydroxycholesterol biosynthesis promotes midbrain dopaminergic neuron development

**DOI:** 10.1016/j.isci.2023.108670

**Published:** 2023-12-07

**Authors:** James Hennegan, Aled H. Bryant, Lauren Griffiths, Matthieu Trigano, Oliver J.M. Bartley, Joanna J. Bartlett, Carys Minahan, Willy Antoni Abreu de Oliveira, Eylan Yutuc, Sotirios Ntikas, Christos S. Bartsocas, Margarita Markouri, Eleni Antoniadou, Ioanna Laina, Owain W. Howell, Meng Li, Yuqin Wang, William J. Griffiths, Emma L. Lane, Mariah J. Lelos, Spyridon Theofilopoulos

**Affiliations:** 1 Regenerative Neurobiology Laboratory, Swansea University Medical School, Institute of Life Science 1, Singleton Park, Swansea SA2 8PP, UK; 2 Brain Repair Group, School of Biosciences, Cardiff University, Cardiff CF10 3AX, UK; 3 Oxysterol Research Group, Swansea University Medical School, ILS1 Building, Singleton Park, Swansea SA2 8PP, UK; 4 Multiple Sclerosis Research Group, Swansea University Medical School, ILS1 Building, Singleton Park, Swansea SA2 8PP, UK; 5 Dementia Research Institute, Cardiff University, Hadyn Ellis Building, Cardiff CF24 4HQ, UK; 6 School of Pharmacy and Pharmaceutical Sciences, Cardiff University, Cardiff CF10 3NB, UK; 7 Laboratory of Molecular Neurobiology, Department of Medical Biochemistry and Biophysics, Karolinska Institutet, 17177 Stockholm, Sweden; 8 Athens Medical Center, 15125 Athens, Greece; 9 Neuroscience and Mental Health Innovation Institute, Cardiff University, Cardiff CF24 4HQ, UK; 10 MRC Centre for Neuropsychiatric Genetics and Genomics, School of Medicine, Cardiff University, Cardiff CF24 4HQ, UK

**Keywords:** Biological sciences, neuroscience, Molecular neuroscience

## Abstract

Dysregulated cholesterol metabolism has been linked to neurodegeneration. We previously found that free, non-esterified, 7α,(25*R*)26-dihydroxycholesterol (7α,26-diHC), was significantly elevated in the cerebrospinal fluid of patients with Parkinson’s disease (PD). In this study we investigated the role of 7α,26-diHC in midbrain dopamine (mDA) neuron development and survival. We report that 7α,26-diHC induces apoptosis and reduces the number of mDA neurons in hESC-derived cultures and in mouse progenitor cultures. Voriconazole, an oxysterol 7α-hydroxylase (CYP7B1) inhibitor, increases the number of mDA neurons and prevents the loss of mDA neurons induced by 7α,26-diHC. These effects are specific since neither 7α,26-diHC nor voriconazole alter the number of Islet1^+^ oculomotor neurons. Furthermore, our results suggest that elevated 24(*S*),25-epoxycholesterol, which has been shown to promote mDA neurogenesis, may be partially responsible for the effect of voriconazole on mDA neurons. These findings suggest that voriconazole, and/or other azole CYP7B1 inhibitors may have implications in PD therapy development.

## Highlights

• 7α,26-dihydroxycholesterol induces apoptosis of midbrain dopamine (mDA) neurons
• CYP7B1 inhibition increases the number of mDA neurons and prevents their loss
• There is no effect on the number of Islet1^+^ oculomotor neurons
• Elevated 24,25-epoxycholesterol may be responsible for the effect of voriconazole

## Graphical abstract

**Figure:**
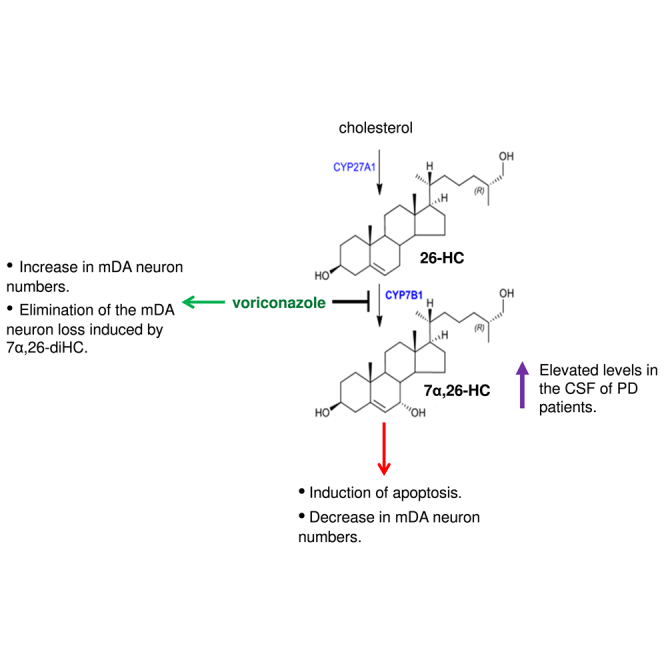

## Introduction

About 25% of total body cholesterol can be found in the brain, and the substantia nigra pars compacta (SNc), where midbrain dopamine (mDA) neurons reside, is particularly rich in cholesterol.^1^^,^^2^ Specific cholesterol metabolites (oxysterols) have been shown to play critical roles in the development and maintenance of mDA neurons. For example, the activation of liver X receptors LXRα/*Nr1h3* and LXRβ/*Nr1h2* by 22(*R*)-hydroxycholesterol resulted in increased mDA yield in mouse embryonic stem cell (ESC) culture and mouse VM progenitor culture.^3^^,^^4^^,^^5^ Additionally, endogenous 24(*S*),25-epoxycholesterol (24,25-EC) has been shown to selectively promote mDA neurogenesis *in vitro* and *in vivo* in the developing mouse midbrain.^6^^,^^7^ Furthermore, it has been shown that the effect of oxysterols and related compounds on mDA neurogenesis can be blocked *in vivo* by inhibiting the basic-helix-loop-helix transcription factor sterol regulatory element-binding protein 1 (SREBP1; gene *Srebf1*), showing that *Srebf1* is a central component of the transcriptional network controlling mDA neurogenesis.^8^ Taken together, these data support the concept that specific oxysterols play critical roles in mDA development and survival.

Furthermore, certain cholesterol metabolites may have detrimental effects on mDA neuron maturation and/or survival, as previously observed in the case of specific cholestenoic acids and Islet1^+^ oculomotor neurons.^9^ In Parkinson’s disease (PD), the second most common progressive degenerative disorder of the central nervous system,^10^ the mDA neurons of the SNc that project to the striatum, among other neuronal types, degenerate. This has been associated with the motor (bradykinesia, resting tremor, rigidity) and some non-motor (cognitive, sensory and sleep abnormalities, depression) symptoms of PD.^10^^,^^11^^,^^12^ Dysregulated cholesterol metabolism has been linked to PD, as well as to a number of other neurodegenerative conditions.^13^^,^^14^^,^^15^^,^^16^^,^^17^^,^^18^ We have previously shown that free, non-esterified 7α,(25*R*)26-dihydroxycholesterol (7α,26-diHC) is significantly elevated in the CSF of patients with PD compared to control individuals and suggested that the acidic pathway of bile acid biosynthesis is dysregulated in the central nervous system of patients with PD.^2^ Based on these findings, we explore in this study the effect of 7α,26-diHC on mouse and human mDA survival, and we utilise 7α,26-diHC biosynthetic inhibitors to determine whether they affect mDA neuron development.

Essentially all brain cholesterol is synthesized *in situ* in the brain.^1^^,^^19^ Cholesterol is metabolized in the brain by the neuron-specific cytochrome P450 family 46 subfamily A member 1 (CYP46A1, also known as cholesterol 24S-hydroxylase) to 24(*S*)-hydroxycholesterol (24-HC), which can cross the blood–brain barrier (BBB) and enter the circulation.^19^^,^^20^^,^^21^ Cells in peripheral organs preferentially convert cholesterol by CYP27A1 (sterol 27-hydroxylase) to (25*R*)26-hydroxycholesterol (26-HC), also known by the non-systematic name 27-hydroxycholesterol,^22^ which can cross the BBB and enter the brain from the circulation.^23^^,^^24^ 26-HC in the brain is converted to 7α,26-diHC (also known as 7α,27-diHC and cholest-5-ene-3β,7α,(25*R*)26-triol) by CYP7B1 (oxysterol 7α-hydroxylase).^2^^,^^24^^,^^25^^,^^26^^,^^27^^,^^28^ 7α,26-diHC has been shown to be a low-affinity agonist of the retinoic acid receptor-related orphan receptor gamma (RORγ; gene *RORC*) and gamma t (RORγt) in human embryonic kidney cells.^29^ Certain CYP7B1 inhibitors, such as the non-azole molecule metyrapone and the azole molecule voriconazole have been shown to interact with and significantly inhibit brain CYP7B1^28^ and therefore would presumably inhibit endogenous 7α,26-diHC biosynthesis from cholesterol.

The aims of the present study were the following: Firstly, to explore whether 7α,26-diHC affects mDA development or survival; secondly, to determine whether the inhibition of 7α,26-diHC biosynthesis affects the number of mDA neurons in mouse and human cultures, and thirdly, to determine whether 7α,26-diHC is upregulated in the α-synuclein mouse model of PD.

To address these aims, we studied the effects of 7α,26-diHC, as well as of the biosynthetic inhibitors metyrapone and voriconazole, in cultures of VM ReNcell cells, mouse progenitor midbrain cells and RC17 human ESCs. We also measured levels of specific cholesterol metabolites in wild-type mice and mice with the viral overexpression of A53T α-synuclein.

## Results

### The CYP7B1 inhibitor voriconazole increases the number of TH^+^ neurons and rescues their loss by 7α,26-dihydroxycholesterol

**Figure 1:**
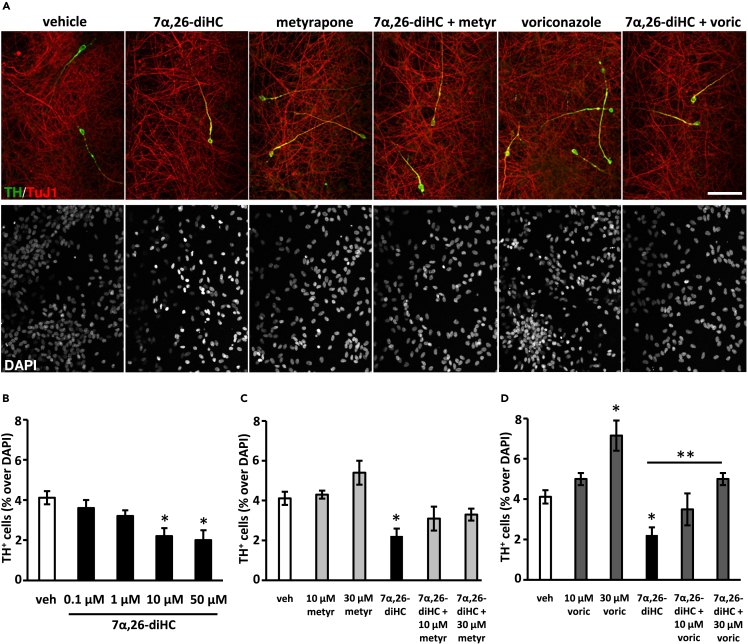
Decreased number of tyrosine hydroxylase-positive neurons in human ReN midbrain cultures treated with 7α,26-diHC and elimination of the toxic effect by voriconazole (A) Representative images of TH^+^ and TuJ1^+^ neurons, as well as DAPI^+^ nuclei, in cultures treated with 30 μM metyrapone, 30 μM voriconazole and/or 10 μM 7α,26-diHC. (B) Quantification of TH^+^ neurons relative to the total DAPI^+^ cells in cultures treated with increasing concentrations of 7α,26-diHC. (C and D) Quantification of TH^+^ neurons relative to the total DAPI^+^ cells in cultures treated with metyrapone (C), voriconazole (D) and/or 10 μM 7α,26-diHC at the indicated concentrations and combinations. Data represent mean ± SEM (n = 3–4); ∗p < 0.05, ∗∗p < 0.01, by one-way ANOVA test, compared to vehicle treatment, or as indicated. Scale bar, 20 μm.

As it has previously been shown that 7α,26-diHC is elevated in CSF of patients with PD, we set out to study the effect of 7α,26-diHC on dopaminergic neurons. We first utilised an immortalised human neural progenitor ventral midbrain cell line (VM ReN cells) which is capable of giving rise to mDA neurons.^30^ Treatment of VM ReN cells with 7α,26-diHC during the latter seven days of the differentiation period, resulted in a dose-dependent decrease in the number of tyrosine hydroxylase positive neurons (TH, the rate-limiting enzyme in the synthesis of dopamine). 10–50 μM 7α,26-diHC significantly reduced the number of TH^+^ neurons compared to vehicle treatment; the number of TH^+^ neurons after treatment with 10 μM 7α,26-diHC was 53% of that after vehicle treatment (Figures 1A and 1B). These neurons co-expressed TH and TuJ1 (β III tubulin, a pan-neuronal marker).

To reduce the endogenous levels of 7α,26-diHC, that can be formed from cholesterol in the cultures, we subsequently treated the cultures with two CYP7B1 inhibitors, the non-azole molecule metyrapone and the azole molecule voriconazole, both of which have been shown to interact with and significantly inhibit brain CYP7B1.^28^ Voriconazole, but not metyrapone, also inhibits cytochrome P450 14α-sterol demethylase (CYP51), a key enzyme in the Bloch and Kandustch-Russell pathways of cholesterol biosynthesis, which demethylates lanosterol and 24,25-dihydrolanosterol.^31^ Analysis of the developing VM by single-cell RNA-Seq^32^^,^^33^ revealed that *Cyp7b1* and *CYP7B1* were expressed in several mouse and human VM cell types, with highest expression in three cell types lining the ventricle, mouse ependymal cells (mEpend), as well as mouse radial glia-like cells 2 and 3 (mRgl2 and mRgl3) (Figure S1A). *Cyp51* was also highly expressed in Rgl2 cells of the developing VM (Figure S1B). Treatment of VM ReN cells with metyrapone did not have any significant effect on the number of TH^+^ neurons compared to vehicle, and did not significantly rescue the loss of TH^+^ neurons induced by 7α,26-diHC, but cultures receiving both 7α,26-diHC and metyrapone also did not show any significant reduction compared to vehicle, suggesting a partial effect by metyrapone (Figures 1A and 1C). To further examine this possibility, we treated the cultures with voriconazole and found a significant 76% increase in the number of TH^+^ neurons compared to vehicle after treatment with 30 μM voriconazole (Figures 1A and 1D). Moreover, 30 μM voriconazole significantly increased the number of TH^+^ neurons in cultures treated with both 7α,26-diHC and voriconazole, relative to cultures treated with 7α,26-diHC alone.

**Figure 2:**
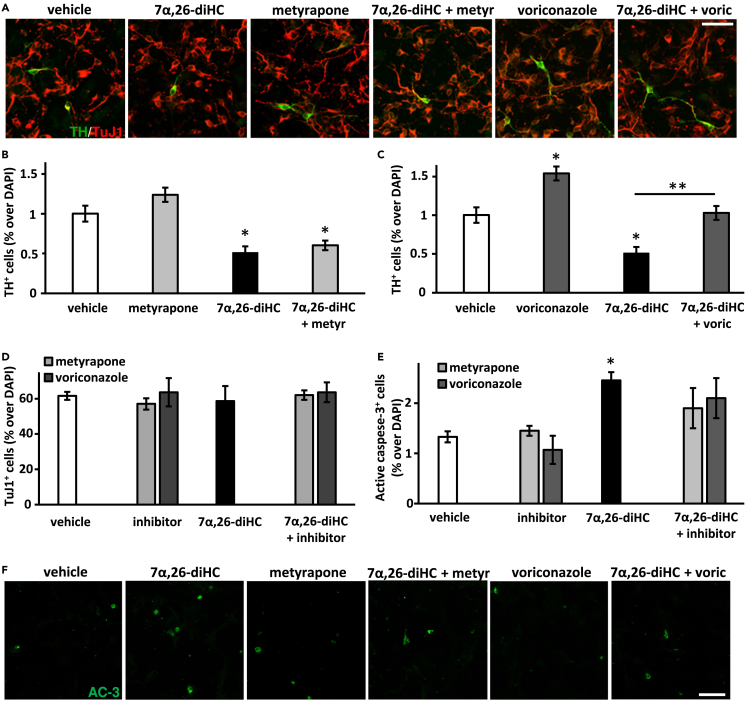
Differential effects by 30 μM metyrapone, 30 μM voriconazole and 10 μM 7α,26-diHC on mDA neuron development and cell survival in mouse ventral midbrain progenitor cultures (A) Representative images of TH^+^ and TuJ1^+^ neurons in cultures treated with voriconazole, metyrapone and/or 7α,26-diHC. (B and C) Quantification of TH^+^ neurons relative to the total DAPI^+^ cells in cultures treated with metyrapone (B), voriconazole (C) and/or 7α,26-diHC. (D) Quantification of TuJ1^+^ neurons relative to the total DAPI^+^ cells in cultures treated with metyrapone, voriconazole and/or 7α,26-diHC. (E) Quantification of active caspase-3^+^ cells relative to the total DAPI^+^ cells in cultures treated with metyrapone, voriconazole and/or 7α,26-diHC. (F) Representative images of active caspase-3^+^ (AC-3^+^) cells in cultures treated with metyrapone, voriconazole and/or 7α,26-diHC. Data represent mean ± SEM (n = 3–4); ∗p < 0.05, ∗∗p < 0.01, by one-way ANOVA test, compared to vehicle treatment, or as indicated. Scale bars, 20 μm.

Similar results were obtained in mouse VM progenitor cultures. Treatment with 7α,26-diHC significantly reduced the number of TH^+^ neurons in the cultures by 49% compared to vehicle treatment (Figures 2A, 2B, and 2C). Voriconazole, but not metyrapone, increased by 54% the number of TH^+^ neurons compared to vehicle. Voriconazole also significantly increased the number of TH^+^ neurons in cultures treated with both 7α,26-diHC and voriconazole, relative to cultures treated with 7α,26-diHC alone. These neurons co-expressed TH, the pan-neuronal marker TuJ1 (β III tubulin), pituitary homeobox 3 (Pitx3, a transcription factor required for the survival and maintenance of mDA neurons^34^) and vesicular monoamine transporter 2 (Vmat2, that is required for the transport of dopamine from the cytosol to synaptic vesicles^35^) (Figures 2A and S2), thereby showing that the derived TH^+^ neurons exhibited a phenotype consistent with that of mDA neurons. Additionally, we did not observe any significant change in the total number of TuJ1^+^ neurons, that represent the majority of cells in culture, by any of the treatments (Figures 2A and 2D). We hypothesised that the changes induced by 7α,26-diHC in these cultures could result from the regulation of cell survival or death and examined the number of cells undergoing apoptosis as assessed by active (cleaved) caspase-3 immunoreactivity. Treatment with 7α,26-diHC, but not the other compounds studied, significantly increased the number of active caspase-3^+^ cells, and therefore apoptosis, by 84% in mouse VM progenitor cultures (Figures 2E and 2F).

### No effect on the number of Islet1^+^ neurons in midbrain progenitor cultures treated with the CYP7B1 inhibitor voriconazole

**Figure 3:**
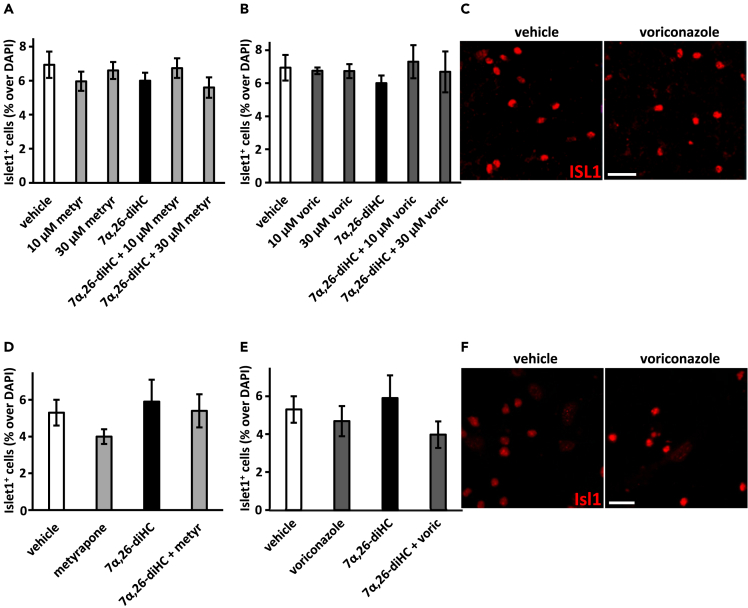
No effect on Islet1^+^ oculomotor neurons in human ReN midbrain cultures or in mouse ventral midbrain progenitor cultures treated with metyrapone or voriconazole and/or 7α,26-diHC (A and B) Quantification of Islet1^+^ cells relative to the total DAPI^+^ cells in human ReN midbrain cultures treated with metyrapone (A), voriconazole (B) and/or 10 μM 7α,26-diHC at the indicated concentrations and combinations. (C) Representative images of Islet1^+^ cell nuclei in human ReN midbrain cultures treated with vehicle or 30 μM voriconazole. (D and E) Quantification of Islet1^+^ cells relative to the total DAPI^+^ cells in mouse ventral midbrain progenitor cultures treated with 30 μM metyrapone (D), 30 μM voriconazole (E) and/or 10 μM 7α,26-diHC. (F) Representative images of Islet1^+^ cell nuclei in mouse ventral midbrain progenitor cultures treated with vehicle or 30 μM voriconazole. Data represent mean ± SEM (n = 3–4). Scale bars, 20 μm.

In human VM ReN cell cultures, no significant change in the number of Islet1^+^ oculomotor neurons was detected by the compounds of interest at any of the concentrations and combinations used (Figures 3A, 3B, and 3C), thereby suggesting that the effects of 7α,26-diHC and voriconazole we previously observed in these cultures were specific to TH^+^ neurons. In order to undeniably verify this suggestion, the effect of the compounds of interest should be studied and analyzed on all different cell types present in culture.

Similar results were obtained in mouse VM progenitor cultures. We did not observe any significant change in the number of Islet1^+^ oculomotor neurons by any of the treatments (Figures 3D, 3E, and 3F), thereby again suggesting that the effects of 7α,26-diHC and voriconazole we previously observed in these cultures were specific to TH^+^ neurons.

### Inhibiting the biosynthesis of toxic 7α,26-dihydroxycholesterol by voriconazole results in elevated number of midbrain dopamine neurons derived from human embryonic stem cells

**Figure 4:**
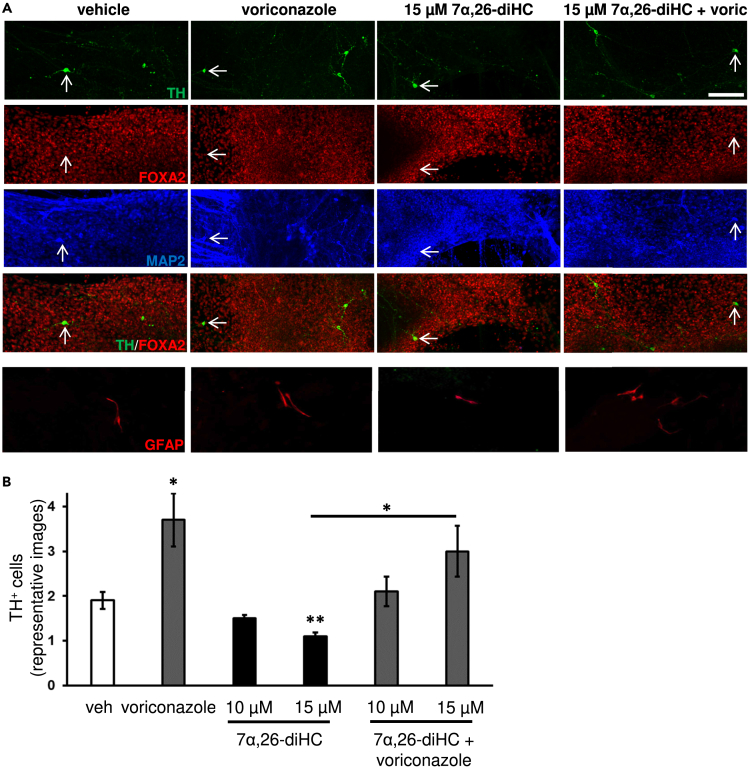
Decreased number of hESC-derived tyrosine hydroxylase-positive neurons in cultures treated with 15 μM 7α,26-diHC and elimination of the toxic effect by 30 μM voriconazole (A) Representative images of hESC-derived TH^+^ and MAP2^+^ neurons, FOXA2^+^ cell nuclei, as well as GFAP^+^ glial cells in cultures treated with voriconazole and/or 7α,26-diHC. Scale bar, 40 μm. (B) Quantification of hESC-derived TH^+^ neurons from representative images of cultures treated with voriconazole and/or 7α,26-diHC. Data represent mean ± SEM (n = 3–4); ∗p < 0.05, ∗∗p < 0.01, by one-way ANOVA test, compared to vehicle treatment, or as indicated.

We subsequently studied the effect of the compounds of interest on the development of mDA neurons derived from RC17 hESCs as described previously.^36^ Treatment of these cells with the compounds of interest from D25 until the end of the culturing period resulted in very few cells expressing the glial-cell marker glial fibrillary acidic protein (GFAP) at the end of the differentiation period, whilst the majority of cells expressed the pan-neuronal marker microtubule-associated protein 2 (MAP2) (Figure 4A). Under basal conditions, a proportion of MAP2^+^ neurons also expressed LIM homeobox transcription factor 1 alpha (LMX1A, a transcription factor that is required for the specification of mDA neurons^37^) (Figure S3). Further immunocytochemical analysis revealed the expression of Forkhead box protein A2 (FOXA2, a transcription factor that is involved in the regulation of midbrain development^38^) and MAP2 in hESC-derived TH^+^ cells (Figure 4A), thereby showing that TH^+^ neurons derived from hESCs using this protocol exhibited a phenotype consistent with that of mDA neurons. Although the proportion of vehicle-treated and 7α,26-diHC and/or voriconazole-treated TH^+^ cells that co-expressed FOXA2 was very similar (data not shown), the number of TH^+^ cells co-expressing FOXA2 was greater in voriconazole–treated cultures (Figures 4A and 4B). Treatment with 15 μM 7α,26-diHC significantly reduced by 42% the number of TH^+^ cells in culture compared to vehicle treatment (Figures 4A and 4B). Voriconazole increased by 95% the number of TH^+^ neurons compared to vehicle. Voriconazole also increased the number of TH^+^ neurons in cultures treated with both 15 μM 7α,26-diHC and voriconazole, relative to cultures treated with 15 μM 7α,26-diHC alone.

Combined, our results thus far demonstrate that voriconazole specifically promotes the development of mDA neurons in human and mouse cultures and that this effect is plausibly due to the biosynthetic inhibition of toxic 7α,26-diHC.

### Increased levels of 24(*S*)-hydroxycholesterol, but not of 7α,26-dihydroxycholesterol, in plasma of α-synuclein-injected mice

In order to study whether similar changes in levels of specific sterols, oxysterols and cholestenoic acids were observed in a rodent model of PD,^39^^,^^40^ compared to what we have previously observed in CSF of patients with PD,^2^ we measured the concentration of the aforementioned compounds in plasma of α-synuclein-injected mice. Six weeks after the intranigral administration of α-synuclein-harbouring viral vectors, mice exhibited unilateral expression of human α-synuclein throughout the SNc and into the striatum, as well as a decrease of TH expression in the striatum, although not reaching statistical significance (Figure S4). Analysis of oxysterol levels in plasma of α-synuclein-injected mice showed that the concentration of 24(*S*)-hydroxycholesterol was 19% higher in mice injected with the high titer α-synuclein compared to empty vector-injected mice (Table S1), but the levels of other oxysterols and cholestenoic acids studied, including 7α,26-diHC, were similar in α-synuclein- and vector-injected mice.

## Discussion

In the present study we report that 7α,26-diHC, an oxysterol that has been shown to be significantly elevated in CSF of patients with PD, induces apoptosis in mouse midbrain progenitor cultures and reduces the number of mDA neurons in hESC-derived cultures and in mouse progenitor cultures. Inhibiting the biosynthesis of 7α,26-diHC by the CYP7B1 and Cyp7b1 inhibitor voriconazole, specifically results in elevated number of mDA neurons in human and mouse cultures. Moreover, voriconazole prevents the loss of mDA neurons induced by 7α,26-diHC, either by increasing the yield of mDA neurons relative to 7α,26-diHC-only treatment or by inhibiting 7α,26-diHC biosynthesis.

The inhibition of CYPB1 by voriconazole may have broader effects, beyond modulating the biosynthesis of 7α,26-diHC. Like 26-HC, 24,25-EC is also a substrate for CYP7B1 (Figure S5) and it has previously been shown that 24,25-EC levels are significantly elevated in *Cyp7b1*^*−/−*^ mouse brain.^41^^,^^42^ Endogenous 24,25-EC has been shown to selectively promote mDA neurogenesis *in vitro* and *in vivo* in the developing mouse midbrain^6^^,^^7^ suggesting that elevated 24,25-EC, due to voriconazole-induced CYP7B1 inhibition, could be contributing to the increase in mouse mDA neurons we observe in our current study. Additionally, we have shown that total 24,25-EC levels are about 8-fold higher in plasma of patients with hereditary spastic paraplegia type 5 (SPG5, caused by biallelic mutations in *CYP7B1*),^9^^,^^43^^,^^44^ compared to levels representative of the population of the USA (i.e., National Institute of Standards and Technology Standard Reference Material 1950 [NIST SRM 1950])^45^ (Table S2), suggesting that 24,25-EC could also be contributing to the increase in human mDA neurons we observe in our current study after inhibiting CYP7B1 by voriconazole. Interestingly, there are no reports of Parkinson’s disease in patients with SPG5.

We have also previously shown that other oxysterols and cholestenoic acids that exhibit altered levels in *Cyp7b1*^*−/−*^ mouse brain, i.e., 25-hydroxycholesterol (25-HC), 26-HC and 3β-hydroxycholestenoic acid (3β-HCA),^41^^,^^42^ as well as in the CSF and plasma of patients with SPG5, i.e., 25-HC, 26-HC, 3β-HCA and 3β,7α-dihydroxycholestenoic acid (3β,7α-diHCA),^9^ do not have an effect on the number of mouse mDA neurons *in vitro* and, for 3β-HCA and 3β,7α-diHCA, also *in vivo*.^6^^,^^9^ The aforementioned data suggests that the increase in the number of mDA neurons we observe in the present study by voriconazole, is due to the elimination of the toxic effect of 7α,26-diHC, as well as, presumably, due to the promotion of the neurogenic effect of 24,25-EC.

As discussed previously, analysis of the developing VM by single-cell RNA-Seq^32^^,^^33^ has revealed that *Cyp7b1* and *Cyp51* are highly expressed in mRgl2 cells (Figure S1). Interestingly, *in situ* hybridization analysis in the developing mouse VM,^5^ as well as transcriptome profile analysis of mRgl2 cells^46^ has revealed an enrichment in the expression of genes related to cholesterol biosynthesis and catabolism. These include 2,3-oxidosqualene-lanosterol cyclase, *Osc*, also known as lanosterol synthase, *Lss* (Figure S6), that is involved in the biosynthesis of cholesterol and 24,25-EC via the shunt pathway which runs in parallel to the cholesterol biosynthesis pathway (Figure S5), as well as the basic-helix-loop-helix transcription factor sterol regulatory element-binding protein 1 (SREBP1; gene *Srebf1*), a master regulator of *de novo* lipogenesis.^8^ Moreover, *Cyp46a1* and *CYP46A1* are highly expressed in Rgl2 and Rgl3 cells^7^; CYP46A1 can generate 24,25-EC, a CYP7B1 substrate, from desmosterol (Figure S5). These observations possibly link VM mRgl2 cells to the production of specific sterols and oxysterols controlling mDA neurogenesis and survival.

Another interesting observation in our study is that voriconazole had a significant effect in elevating the number of mDA neurons in mouse and human midbrain cultures, whereas metyrapone did not. Both molecules interact with and significantly inhibit brain CYP7B1.^28^ Additionally, voriconazole inhibits brain CYP46A1, which catalyses the formation of 24-HC and 24,25-EC,^7^^,^^19^^,^^20^^,^^21^ by the direct binding and inhibition of CYP46A1.^47^ As mentioned earlier, voriconazole also inhibits CYP51, which is highly expressed in VM Rgl2 cells^32^^,^^33^ (Figure S1B). Reduced activity of both CYP46A1 and CYP51 could lead to a reduced rate of synthesis of brain cholesterol and reduced the formation of specific cholesterol metabolites (including toxic 7α,26-diHC).

On the other hand, metyrapone inhibits enzymes of the CYP11B and CYP2B families, which include 11beta-hydroxylase (CYP11B1) and aldosterone synthase (CYP11B2), that are involved in the biosynthesis of cortisol from 11-deoxycortisol and of aldosterone from 11-deoxycorticosterone.^48^^,^^49^ Members of the aforementioned enzyme families are expressed in Rgl2 cells of the mouse and human developing VM,^32^^,^^33^ cells that are, as mentioned earlier, linked to the production of specific sterols and oxysterols controlling mDA neurogenesis and survival. We hypothesize that the inhibition of biosynthesis of multiple steroids by metyrapone in mouse and human cells could have additional effects on the differentiation or survival of certain cell types in culture and could thus result in non-significant effects on the number of mDA neurons. An alternative explanation could be that metyrapone, under our culturing conditions, is not stable in solution and that is why we do not observe any significant effect in our cultures.

Furthermore, neither 7α,26-diHC nor voriconazole had an effect on the number of Islet1^+^ oculomotor neurons in mouse and human midbrain cultures, adding further support toward the specificity of effects by certain oxysterols on different VM neuronal types we have described earlier.^6^^,^^9^ An alternative explanation would be that oculomotor neurons do not synthesize 7α,26-diHC, therefore treatment with voriconazole would not have an effect on this neuronal type.

Moreover, we have observed that 24(S)-hydroxycholesterol (24-HC), the most abundant oxysterol in the adult mammalian brain,^19^ has no effect on the number of mDA progenitors and mDA neurons derived from mouse midbrain progenitor cultures or from hESCs, compared to vehicle treatment (ref.^7^ and data not shown). This provides further evidence toward the specificity of effects by different oxysterols in the VM we have described earlier.^6^^,^^9^ Interestingly, we found in the current study an increase in 24-HC levels in plasma of α-synuclein-injected mice. It has previously been shown in some, but not in other, studies that 24-HC levels are increased in CSF of patients with PD.^2^^,^^15^ Although we did not find any significant increase in 7α,26-diHC levels in α-synuclein-injected mice, it is worth noting that the levels of 7α,26-diHC were very low, close to the detection limit, leaving open the possibility for further investigation of this oxysterol in rodent models of PD.

The findings presented in this study demonstrate that 7α,26-diHC, which is significantly elevated in CSF of patients with PD, induces apoptosis in mouse midbrain progenitor cultures and reduces the number of mDA neurons in hESC-derived cultures and in mouse progenitor cultures. We suggest that voriconazole, and/or other azole CYP7B1 inhibitors, could be utilized to specifically increase the yield of mDA progenitors and neurons. The fact that elevated levels of 24,25-EC, which has been shown to promote mDA neurogenesis, may be at least partially responsible for the effect of voriconazole on mDA neurons, further suggests that specific azole CYP7B1 inhibitors may be useful in increasing the yield of mDA neurons. Additional studies are needed to investigate the effect of specific azole CYP7B1 inhibitors on mature mDA neurons. Furthermore, *in vivo* studies on the effect of voriconazole, in the presence or absence of 7α,26-diHC, on α-synuclein-injected rodents and/or on 6-hydroxydopamine-injected rodents would further indicate whether voriconazole and/or other azole CYP7B1 inhibitors could increase the number of mDA neurons *in vivo* and whether they could constitute a potential treatment strategy for PD.

### Limitations of the study

The effect of the compounds of interest would need to be further studied on all cell types present in the embryonic VM, including Islet1^+^ oculomotor neurons, Brn3a^+^ red nucleus neurons, GABAergic neurons and serotonergic neurons. Also, the effect of 7α,26-diHC and voriconazole would need to be studied on different neuronal cultures, such as cortical or hippocampal cell cultures. Additionally, further immunocytochemical characterization of the mDA neurons derived in our ReN midbrain cell cultures, mouse midbrain progenitor cell cultures and hESC-derived cultures (for Nurr1, AADC and other protein expression) would be valuable. Furthermore, the effect of additional azole CYP7B1 inhibitors would need to be examined on mDA cultures. Lastly, the effect of voriconazole would need to be studied on mature mDA neurons, as well as in a rodent model of PD in order to determine whether voriconazole could constitute a potential treatment strategy for PD.

## STAR★Methods

### Key resources table

**Table:** 

REAGENT or RESOURCE	SOURCE	IDENTIFIER
**Antibodies**		

anti-α-synuclein	BD Biosciences	Cat # 610786
anti-TH	Millipore	Cat# AB152
anti-TH	Pel-Freez	Cat# P40101-150
anti-TH (185)	Thermo Fisher Scientific - Merck	Cat# MA1-24654
anti-Cleaved Caspase-3 (Asp175)	Cell Signaling	Cat# 9661
anti-Islet 1	Abcam	Cat# ab20670
anti-βIII Tubulin	Promega	Cat# G7121
anti-MAP2	Synaptic Systems	Cat# 188 002
anti-MAP2	Synaptic Systems	Cat# 188 004
anti-GFAP	BioLegend	Cat# 2E1.E9
anti-PITX3/PTX3	Abcam	Cat# ab313403
anti-LMX1A	Merck	Cat# HPA028051
anti-HNF-3beta/FoxA2	Novus Biologicals	Cat# AF2400-SP
anti-VMAT2/SLC18A2	Everest	Cat# EB06558
Goat Anti-Rabbit IgG (H + L), Biotinylated	Vector laboratories	Cat# BA1000
Goat Anti-Mouse IgG (H + L), Biotinylated	Vector laboratories	Cat# BA9200
Goat anti-Rabbit IgG H&L (Alexa Fluor® 488)	Abcam	Cat# ab150077
Goat anti-Mouse IgG H&L (Alexa Fluor® 488)	Abcam	Cat# ab150113
Donkey anti-Rabbit IgG H&L (Alexa Fluor® 488)	Abcam	Cat# ab150073
Donkey anti-Goat IgG H&L (Alexa Fluor® 488)	Abcam	Cat# ab150129
Donkey anti-Goat IgG H&L Cy5	Abcam	Cat# ab6566
Goat anti-Rabbit IgG H&L (Alexa Fluor® 594)	Abcam	Cat# ab150080
Goat anti-Mouse IgG H&L (Alexa Fluor® 594)	Abcam	Cat# ab150116
Donkey anti-Rabbit IgG H&L (Alexa Fluor® 594)	Abcam	Cat# ab150076
Goat anti-Mouse IgG H&L (Alexa Fluor® 647)	Abcam	Cat# ab150115
Goat anti-Rabbit IgG H&L (Alexa Fluor® 647)	Abcam	Cat# ab150079
Donkey anti-Rabbit IgG H&L (Alexa Fluor® 647)	Abcam	Cat# ab150075
Cy2- and Cy3-conjugated secondary antibodies	Jackson ImmunoResearch	

**Bacterial and virus strains**		

Human A53T alpha-synuclein viral vector	Vigene Biosciences	Cat# GD1001-RV
Empty vector control	Vigene Biosciences	Cat# GD1004-RV

**Biological samples**		

Plasma of SPG5 patients	Athens Medical Center, Athens, 15125, Greece	
Plasma of α-synuclein-injected mice	Our experiments at Cardiff University animal facility	

**Chemicals, peptides, and recombinant proteins**		

7α,26-dihydroxycholesterol	Avanti Polar Lipids-Merck	Cat# 700024P
Voriconazole	Sigma-Aldrich	Cat# PZ0005
Metyrapone	Sigma-Aldrich	Cat# M2696

**Critical commercial assays**		

No critical commercial assays were used		

**Deposited data**		

We have not deposited any data		

**Experimental models: Cell lines**		

ReNcell® VM Human Neural Progenitor Cell Line	Merck	Cat# SCC008
RC17 hESC	Roslin Cells	Cat# hPSCreg RCe021-A

**Experimental models: Organisms/strains**		

Female C57Bl6 mice	Charles River, UK	
Male C57BL/6J mice	Cardiff University animal facility	

**Oligonucleotides**		

No oligonucleotides were used		

**Recombinant DNA**		

No recombinant DNA was used		

**Software and algorithms**		

Prism4	GraphPad Software, La Jolla, CA http://www.graphpad.com	
Linnarsson Lab. Gene expression in the human and mouse ventral midbrain.	http://linnarssonlab.org/ventralmidbrain/	

### Resource availability

#### Lead contact

Further information and requests for resources and reagents should be directed to and will be fulfilled by the lead contact, Spyridon Theofilopoulos (s.theofilopoulos@gmail.com).

#### Materials availability

This study did not generate new unique reagents.

#### Data and code availability

Any additional information required to reanalyse the data reported in this paper is available from the lead contact upon request. The data that support the findings of this study are available from the lead contact upon reasonable request.

Neither raw sequencing data nor original code was generated during this work.

### Experimental model and study participant details

Plasma of SPG5 patients was provided by our collaborators in Athens Medical Center, Greece. Plasma of α-synuclein-injected mice was obtained from our experiments at Cardiff University animal facility. All rodent experiments were conducted in compliance with the UK Animals (Scientific Procedures) Act 1986 under Home Office Licence No. P49E8C976 and with the approval of the local Cardiff University Ethics Review Committee.

### Method details

#### Experimental design

To test the hypothesis that 7α,26-diHC and/or inhibitors of its biosynthesis affect mDA development or survival, we studied in Experiment 1 the effect of different concentrations and combinations of 7α,26-diHC, metyrapone and voriconazole in human VM ReNcell cultures by performing immunocytochemical analysis for TH^+^ neurons, TuJ1^+^ neurons and ISL1^+^ neurons. We also utilised a publicly available single-cell RNA-Seq database to analyze the expression pattern of *CYP7B1*, *Cyp7b1*, *CYP51A1*, *Cyp51*, *LSS* and *Lss* in the developing mouse and human VM. Subsequently, we examined, in Experiment 2, the effect of different combinations of 7α,26-diHC, metyrapone and voriconazole on mouse progenitor midbrain cultures and studied by immunocytochemistry TH^+^, Pitx3^+^, VMAT2^+^, Islet1^+^ neurons, as well as TuJ1^+^ neurons and cells undergoing apoptosis (active caspase 3^+^ cells). In Experiment 3, we performed immunocytochemical analysis for TH^+^, LMX1A^+^, FOXA2^+^, MAP2^+^ and GFAP^+^ cells after treatment of RC17 human embryonic stem cell (hESC) cultures with different concentrations and combinations of 7α,26-diHC and voriconazole. Subsequently, in Experiment 4, we studied whether there were elevated 7α,26-diHC levels in a mouse PD model. To address this, we performed α-synuclein viral vector injection in wild-type mice; brains were analyzed by immunohistochemistry for α-synuclein and for TH^+^ neurons, whereas plasma was analyzed by LC-MS for levels of specific oxysterols and cholestenoic acids. Finally, in order to address which oxysterols and cholestenoic acids were altered in patients with hereditary spastic paraplegia type 5 (SPG5, caused by biallelic mutations in *CYP7B1*) relative to what we have previously observed in PD patients, we measured by LC-MS, in Experiment 5, levels of specific oxysterols in plasma of SPG5 patients.

#### Reagents

Authentic sterols and oxysterols were obtained from Avanti Polar Lipids or Merck. Other compounds and consumables were from Merck, Abcam, ThermoFisher Scientific, Greiner, Starlabs, Fujifilm, Miltenyi, Sarstedt, Biolamina or VWR.

#### PreD ReNcell culture

The PredD VM ReNcell culturing protocol described in (Donato et al., 2007) was followed. ReNcell VM cell line is an immortalised stable multipotential human neural progenitor ventral midbrain (VM) line derived from myc overexpression in human primary cells from developing mesencephalon.^30^ Cells were seeded at 30,000 cells/cm^2^ on uncoated 96-well plates in proliferation medium (Advanced DMEM/F12, 1% Penicillin-Streptomycin, 4 mM glutamine, 2% B27 supplement, 50 μg/mL gentamycin, 50 μg/mL heparin, 20 ng/mL bFGF and 20 ng/mL EGF) for 7 days for cell aggregate formation. Cell aggregates were mechanically dissociated by gentle trituration and re-plated on laminin-coated chamber slides. The cells were expanded to confluency in proliferation medium over a 3–4-day period. Differentiation was initiated by removing the proliferation medium and replacing with differentiation medium (same composition as the proliferation medium but without bFGF and EGF) for 7 days. Following this differentiation period, cells were treated with voriconazole, metyrapone and/or 7α,26-diHC at specific concentrations and combinations for 7 days and then fixed for immunocytochemical analysis. 7α,26-diHC, voriconazole, and metyrapone were diluted in 0.1% DMSO to 10 mM and stored at −20 C in aliquots. Each aliquot was diluted to the final concentration in cell culture media on the day that each compound was added to cell cultures.

#### Wild-type mice

Pregnant female C57Bl6 mice (Charles River, UK) were maintained on a 12:12-h light/dark cycle. All experiments were conducted in compliance with the UK Animals (Scientific Procedures) Act 1986 under Home Office Licence No. P49E8C976 and with the approval of the local Cardiff University Ethics Review Committee.

#### Primary mouse progenitor midbrain culture

Brains from E11.5 mice were obtained, and the ventral midbrain (VM) region was dissected, mechanically dissociated, plated on poly-D-lysine (150,000 cells/cm^2^), and grown in serum-free N2 media consisting of F12/DMEM (1:1 mixture) with 10 ng/mL insulin, 100 μg/mL apo-transferrin, 100 μM putrescine, 20 nM progesterone, 30 nM selenium, 6 mg/mL glucose, and 1 mg/mL BSA. Cells were treated for 2 days *in vitro* with voriconazole, metyrapone and/or 7α,26-diHC at specific concentrations and combinations, fixed with 4% PFA, and processed for staining using appropriate antibodies. DAPI staining was performed by permeabilizing cells with a 0.3% Triton X-100/PBS solution for 5 min followed by incubation with DAPI (SlowFade Gold Antifade; ThermoFisher Scientific) for 10 min.

#### RC17 human embryonic stem cells (hESCs) culture

The culturing protocol described in ref. ^36^ was followed for RC17 hESCs (Roslin Cells, hPSCreg RCe021-A). Cells were treated with voriconazole and/or 7α,26-diHC from D25 until D35, and then fixed for immunocytochemical analysis. In the case of studying GFAP^+^ cell differentiation, cells were cultured and treated with the compounds of interest until D45.

#### Immunocytochemical analysis

Cells were fixed in 4% paraformaldehyde (PFA), washed in PBS and blocked in 5% normal goat or donkey serum/PBS for 1 h at room temperature. Primary antibodies were diluted in PBS (pH 7.4), 0.3% Triton X-100, 1% BSA and incubations were carried out overnight at +4°C or at room temperature for 2 h. The antibodies used were: anti-TH (1:1000; Pel-Freez), anti-TH (1:500; Merck), anti-Islet1 (1:500; Abcam), anti-TuJ1 (1:2,000; Promega), anti-MAP2 (1:1,000; Synaptic Systems), anti-GFAP (1:500; BioLegend), anti-Pitx3 (1:500; Abcam), anti-LMX1A (1:1,000; Merck), anti-FOXA2 (1:100; Novus Biologicals), anti-VMAT2 (1:200; Everest), anti-active caspase 3 (or cleaved caspase 3) (1:400; Asp175, Cell Signaling Technology) and appropriate secondary antibodies (Abcam or Jackson ImmunoResearch). Cells positive for the corresponding marker were counted directly at the microscope at a magnification of 20×. Cells were counted in every well, in eight consecutive fields (going from one side of the well to the other, passing through the center), in three different wells per experiment and in at least three different experiments per condition. In the majority of experiments, positive cell counts were normalized to total number of cells (counted utilizing DAPI-stained nuclei). Random images of the wells were taken for every condition to document the result, and representative pictures were subsequently selected to represent the quantitative data. Images were acquired with a laser scanning confocal microscope (Zeiss LSM 710) using the Zeiss Zen microscopy software.

#### Unilateral A53T α-synuclein injections and immunohistochemistry

Male C57BL/6J mice (6–8 weeks old) were injected with A53T α-synuclein at two different concentrations (low titer: 1.7x10^12^ genome copies per mL; high titer: 5.1x10^12^ genome copies per mL), or with empty vector (all from Vigenebio); stereotaxic surgery was performed on day 1 according to ref. ^39^^,^^40^ (unilateral injection to the right SN: AP, −5.2 mm; ML, −2.0 mm; DV, −7.5 mm). In the case of oxysterol and cholestenoic acid measurement experiments, mice were culled by cervical dislocation six weeks post-surgery, blood was collected and plasma was isolated and frozen at −80°C. In the case of histology experiments, mice were culled six weeks post-surgery with sodium pentobarbital (200 mg/mL), transcardially perfused, fixed with paraformaldehyde, the brains were removed, cryoprotected in 30% w/v sucrose solution and stored for histology. Brains were sliced coronally on a freezing microtome into 30 μm sections. Free floating immunohistochemistry for TH and α-synuclein was conducted as previously described.^50^ In brief, sections were quenched in 3% hydrogen peroxide before blocking with goat serum and exposure to the primary antibody (anti-TH Millipore or anti-α-synuclein BD Biosciences). Biotinylated secondary antibodies (Vector laboratories) were utilised. Nigral TH^+^ cell bodies were quantified by manual counts at the level of the 3^rd^ nerve; relative optical density of striatal TH expression was quantified using ImageJ by subtracting the background levels from the corpus callosum.

#### LC-MS measurements in plasma of SPG5 patients and plasma of α-synuclein-injected mice

Measurement of the concentration of oxysterols and cholestenoic acids by Liquid Chromatography-Mass spectrometry (LC-MS) was performed as previously described in ref.^9^ Briefly, sterols were extracted from plasma into ethanol and fractionated by reversed phase solid phase extraction to give a cholestenoic acid- and oxysterol-rich fraction devoid of cholesterol. The sterols were charge-tagged with the Girard P reagent-hydrazine. This greatly enhances their response when analyzed by LC-MS. LC-ESI-MS and LC-ESI-MS^n^ were performed using an Ultimate 3000 HPLC system (Dionex) linked to the ESI source of an LTQ-Orbitrap XL or LTQ-Orbitrap Velos (Thermo Fisher) mass spectrometer.

### Quantification and statistical analysis

Cells positive for the corresponding marker were counted directly at the microscope at a magnification of 20×. Cells were counted in every well, in eight consecutive fields (going from one side of the well to the other, passing through the center), in three different wells per experiment and in at least three different experiments per condition. In the majority of experiments, positive cell counts were normalized to total number of cells (counted utilizing DAPI-stained nuclei).

Statistical analyses (one-way Analysis of Variance [ANOVA] test with Fisher’s Least Significant Difference [LSD] post-hoc test) were performed using Prism4 (GraphPad Software, La Jolla, CA; http://www.graphpad.com). p < 0.05 was considered a statistically significant difference (∗), p < 0.01 (∗∗). Data represent mean ± S.E.M.
