# Supplementary material for: Inhibition of 7α,26-dihydroxycholesterol biosynthesis promotes midbrain dopaminergic neuron development
Source: iScience. 2023 Dec 7;27(1):108670. doi: 10.1016/j.isci.2023.108670 (PMC10753067; doi:10.1016/j.isci.2023.108670)
Supplement: Document S1. Figures S1–S6, Tables S1, and S2 [file mmc1.pdf]

iScience, Volume *27*

Supplemental information

Inhibition of 7α,26-dihydroxycholesterol biosynthesis promotes midbrain dopaminergic neuron development

James Hennegan, Aled H. Bryant, Lauren Griffiths, Matthieu Trigano, Oliver J.M. Bartley, Joanna J. Bartlett, Carys Minahan, Willy Antoni Abreu de Oliveira, Eylan Yutuc, Sotirios Ntikas, Christos S. Bartsocas, Margarita Markouri, Eleni Antoniadou, Ioanna Laina, Owain W. Howell, Meng Li, Yuqin Wang, William J. Griffiths, Emma L. Lane, Mariah J. Lelos, and Spyridon Theofilopoulos

SUPPLEMENTAL FIGURE TITLES AND LEGENDS

Figure S1: Expression of *CYP7B1*, *Cyp7b1, CYP51A1 and Cyp51* in the human and mouse ventral midbrain as assessed by single cell RNA-sequencing, related to Figure 1.

(**A**) Three cell types lining the ventricle, mouse ependymal cells (mEpend) as well as mouse radial glia-like cells 2 and 3 (mRgl2 and mRgl3), express higher levels of *Cyp7b1* compared to all other cell types in the embryonic VM. (**B**) Human and mouse radial glia-like cells 2 (hRgl2a and mRgl2) express higher levels of *CYP51A1* and *Cyp51* compared to other cell types in the embryonic VM. *Cyp51* is also highly expressed in mouse mDA2 neurons and Red Nucleus neurons (mRN). For a list of all cell type abbreviations see ref[32].

Figure S2: Tyrosine hydroxylase-positive neurons co-express the typical dopaminergic markers VMAT2 and Pitx3 in mouse ventral midbrain progenitor cultures as examined by double immunocytochemistry, related to Figure 2.

(**A**) Representative images of double-positive TH^+ and $VMAT2^+$ cells in cultures treated with vehicle or 30 µM voriconazole. (**B**) Representative images of TH^+ cells and $Pitx3^+$ cell nuclei in cultures treated with vehicle or 30 µM voriconazole. Scale bars, 20 µm.

Figure S3: A proportion of hESC-derived MAP2$^+$ neurons express LMX1A, related to Figure 4.

Representative images of MAP2$^+$ neurons and LMX1A$^+$ cell nuclei in cultures derived by hESCs under basal conditions. Arrows indicate double MAP2$^+$;LMX1A$^+$ cells. Arrowheads indicate MAP2$^+$ but LMX1A-negative cells. Scale bar, 10 µm.

Figure S4: Quantification of TH expression six weeks after α-synuclein intranigral administration, related to Figure 4.

(**A**) Quantification of TH expression in the mouse striatum by optical density following intranigral administration of α-synuclein-harbouring viral vectors at two different concentrations (low titre: 1.7×10^{12} genome copies per ml; high titre: 5.1×10^{12} genome copies per ml). (**B**) Quantification of the number of TH$^+$ neuronal cell bodies in the mouse substantia nigra (SNc) presented as a percentage of the uninjected side following intranigral administration of α-synuclein-harbouring viral vectors at two different concentrations. Data represent mean ± SEM (*n* = 4-5). (**C, D**) Photomicrographs of the SN stained for TH$^+$ cells in empty vector-injected mice (C) and α-synuclein-injected mice (D).

Figure S5: Simplified scheme showing the biosynthetic pathways of 7α,26-diHC and 24,25-EC, related to Figure 4.

Biosynthetic enzymes are shown in blue. The CYP7B1 inhibitor voriconazole is shown in red. *CYP7A1* is highly expressed in the liver and gallbladder and shows very low expression in other organs; *CYP7B1* is highly expressed in the brain, liver, spleen, as well as in other peripheral organs.

Figure S6: Expression of *LSS* and *Lss* in the human and mouse ventral midbrain as assessed by single cell RNA-sequencing, related to Figure 4.

Mouse radial glia-like cells 2 (mRgl2) express higher levels of *Lss* (lanosterol synthase, also known as 2,3-oxidosqualene-lanosterol cyclase, *Osc*) compared to all other cell types in the embryonic VM. For a list of all cell type abbreviations see ref[32].

Table S1: Concentration of specific sterols, oxysterols and cholestenoic acids, identified and quantified by LC-MS, in plasma of α-synuclein-injected mice, related to Figure 4.

Table S2: Levels of 24-Oxocholesterol, 24,25-Dihydroxycholesterol and total 24*S*,25-epoxycholesterol, identified and quantified by LC-MS, in plasma of patients with hereditary spastic paraplegia type 5 (SPG5), related to Figure 4.

Figure S1

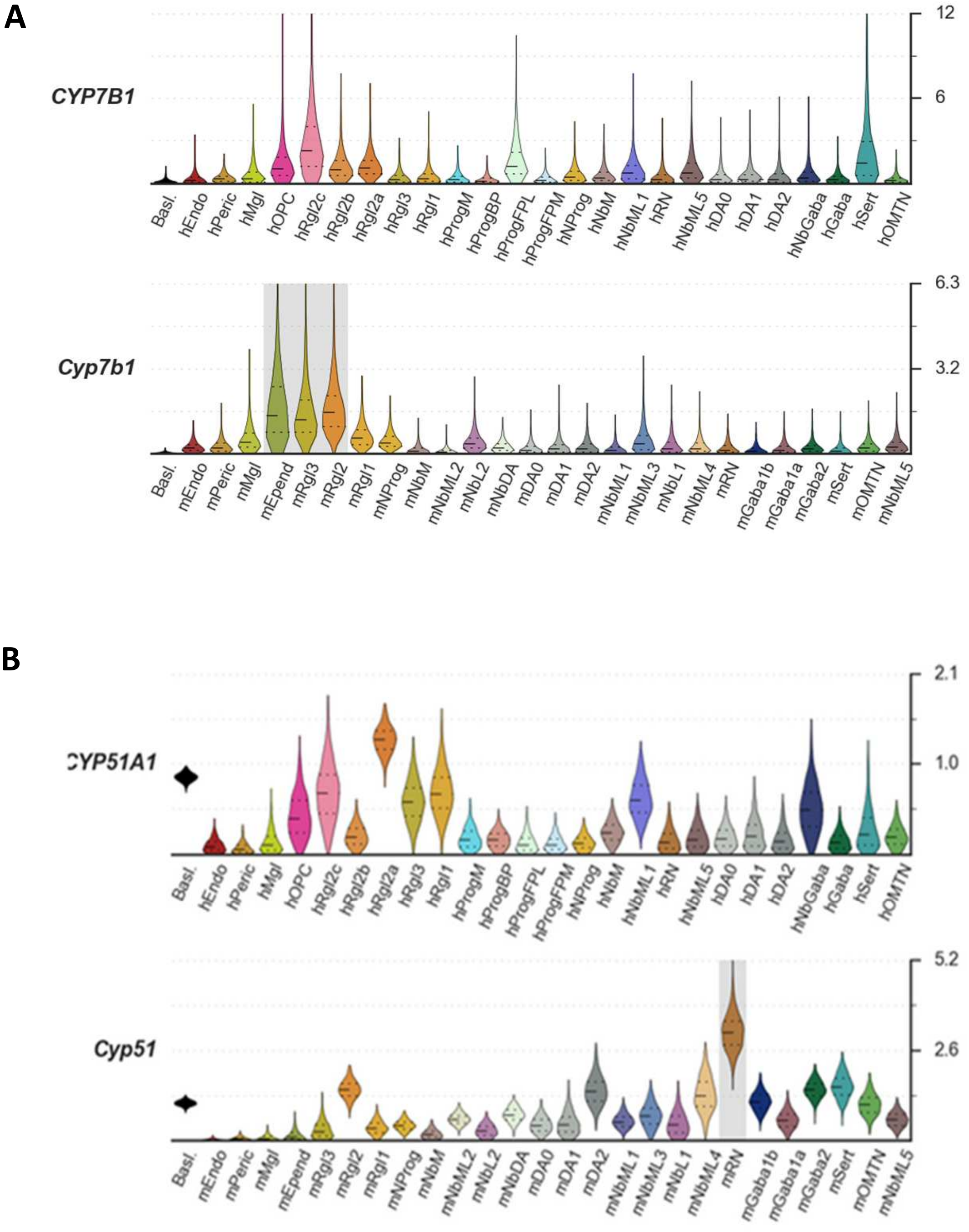
A
CYP7B1
12
6
Basl. hEndo hPeric hMgl hOPC hRgl2c hRgl2b hRgl2a hRgl3 hRgl1 hProgM hProgBP hProgFPL hProgFPM hNProg hNbM hNbML1 hRN hNbML5 hDA0 hDA1 hDA2 hNbGaba hGaba hSert hOMTN
Cyp7b1
6.3
3.2
Basl. mEndo mPeric mMgl mEpend mRgl3 mRgl2 mRgl1 mNProg mNbM mNbML2 mNbL2 mNbDA mDA0 mDA1 mDA2 mNbML1 mNbML3 mNbL1 mNbML4 mRN mGaba1b mGaba1a mGaba2 mSert mOMTN mNbML5
B
CYP51A1
2.1
1.0
Basl. hEndo hPeric hMgl hOPC hRgl2c hRgl2b hRgl2a hRgl3 hRgl1 hProgM hProgBP hProgFPL hProgFPM hNProg hNbM hNbML1 hRN hNbML5 hDA0 hDA1 hDA2 hNbGaba hGaba hSert hOMTN
Cyp51
5.2
2.6
Basl. mEndo mPeric mMgl mEpend mRgl3 mRgl2 mRgl1 mNProg mNbM mNbML2 mNbL2 mNbDA mDA0 mDA1 mDA2 mNbML1 mNbML3 mNbL1 mNbML4 mRN mGaba1b mGaba1a mGaba2 mSert mOMTN mNbML5

Figure S2

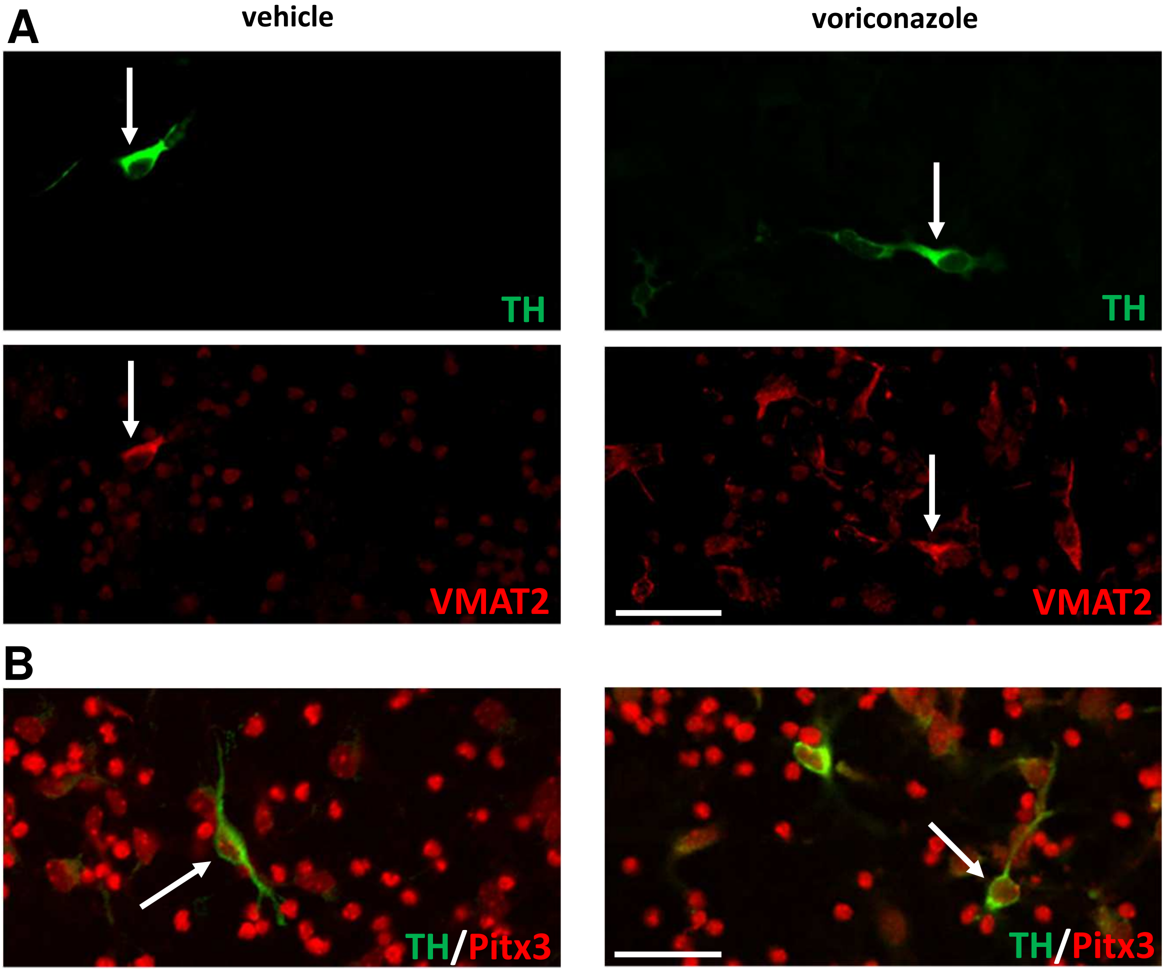

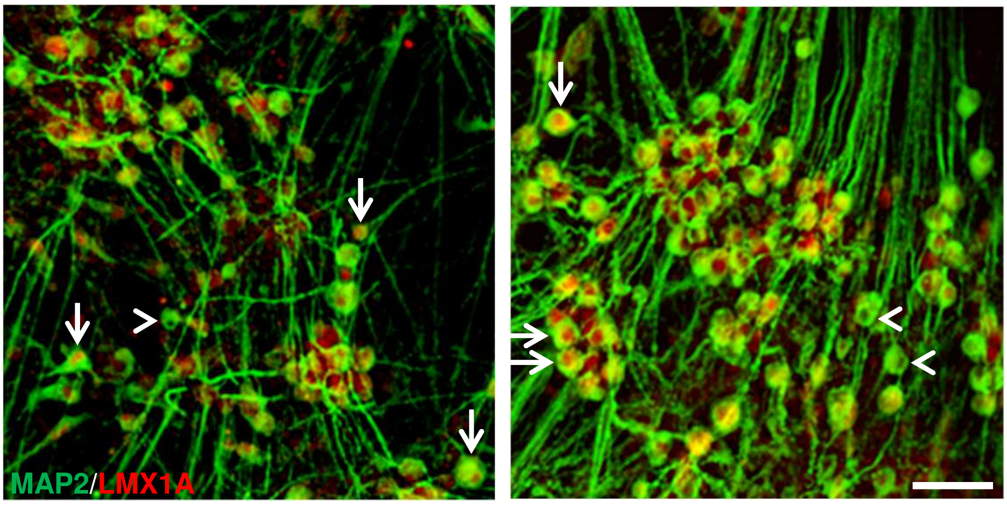
MAP2/LMX1A

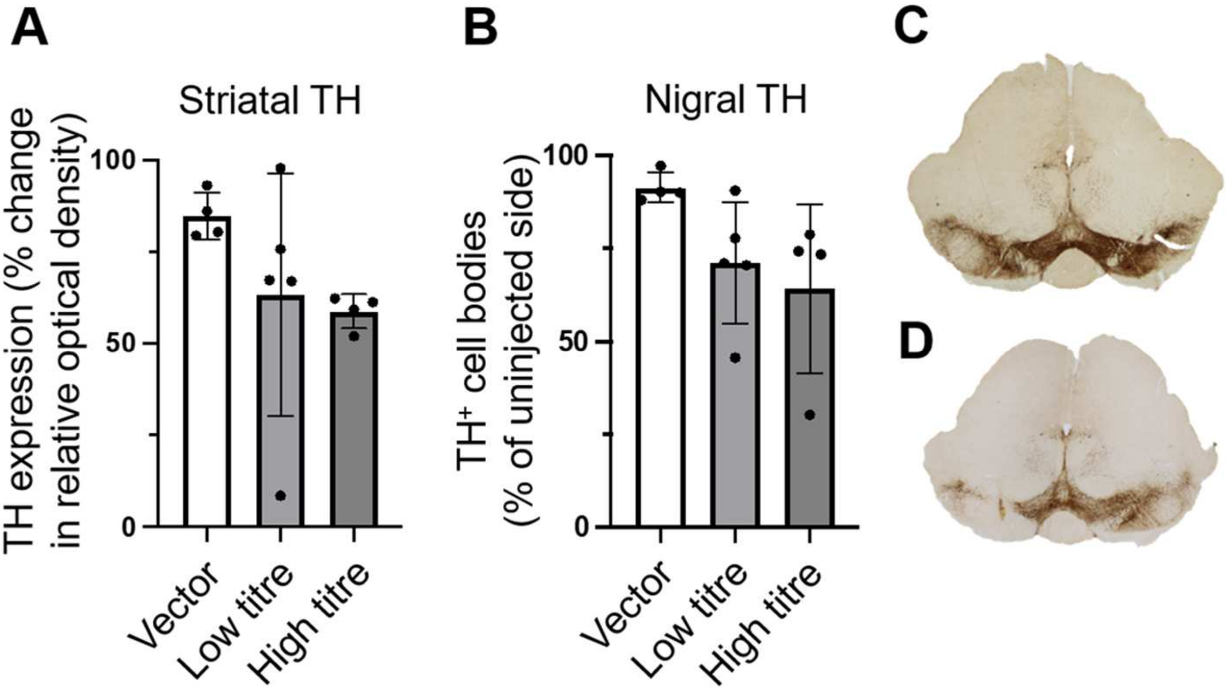
A
Striatal TH
TH expression (% change in relative optical density)
100
50
0
Vector
Low titre
High titre
B
Nigral TH
TH+ cell bodies (% of uninjected side)
100
50
0
Vector
Low titre
High titre
C
D

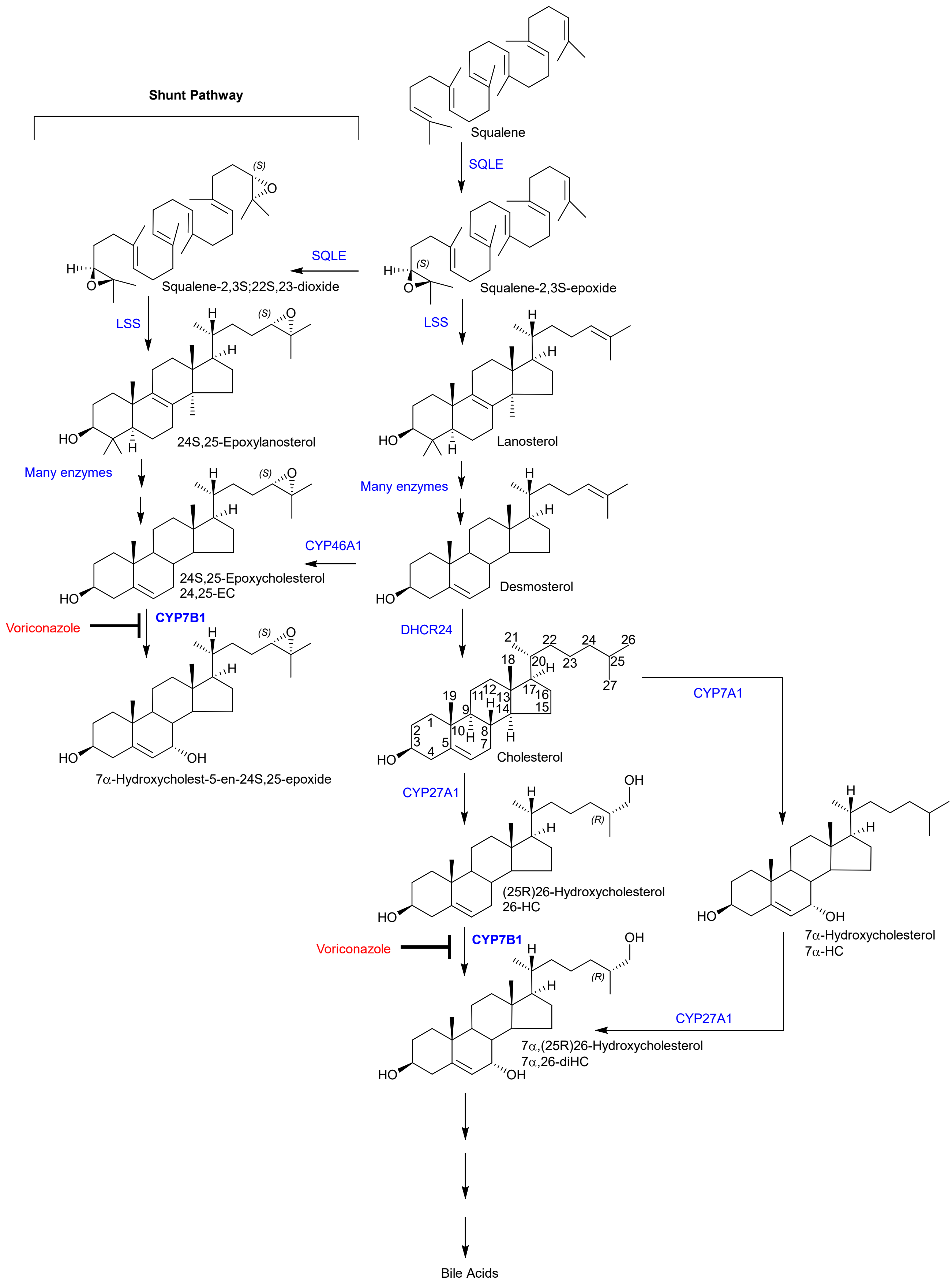
Shunt Pathway
Squalene
SQLE
Squalene-2,3S-epoxide
SQLE
Squalene-2,3S;22S,23-dioxide
LSS
LSS
24S,25-Epoxylanosterol
Lanosterol
Many enzymes
Many enzymes
24S,25-Epoxycholesterol
24,25-EC
CYP46A1
Desmosterol
Voriconazole
CYP7B1
DHCR24
7α-Hydroxycholest-5-en-24S,25-epoxide
Cholesterol
CYP7A1
CYP27A1
(25R)26-Hydroxycholesterol
26-HC
7α-Hydroxycholesterol
7α-HC
Voriconazole
CYP7B1
CYP27A1
7α,(25R)26-Hydroxycholesterol
7α,26-diHC
Bile Acids

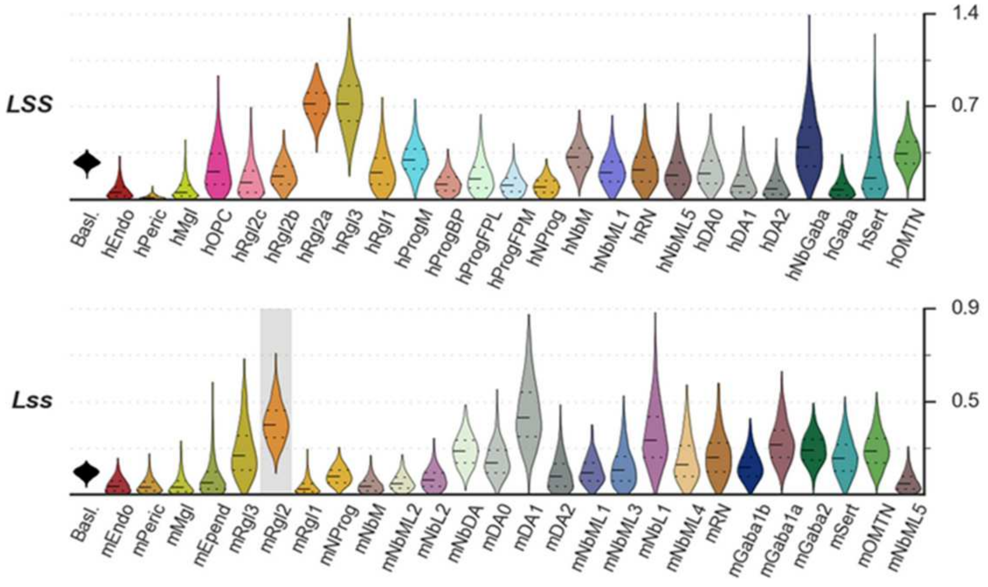
LSS
1.4
0.7
Basl.
hEndo
hPeric
hMgl
hOPC
hRgl2c
hRgl2b
hRgl2a
hRgl3
hRgl1
hProgM
hProgBP
hProgFPL
hProgFPM
hNProg
hNbM
hNbML1
hRN
hNbML5
hDA0
hDA1
hDA2
hNbGaba
hGaba
hSert
hOMTN
Lss
0.9
0.5
Basl.
mEndo
mPeric
mMgl
mEpend
mRgl3
mRgl2
mRgl1
mNProg
mNbM
mNbML2
mNbL2
mNbDA
mDA0
mDA1
mDA2
mNbML1
mNbML3
mNbL1
mNbML4
mRN
mGaba1b
mGaba1a
mGaba2
mSert
mOMTN
mNbML5

Table S1. Concentration of specific sterols, oxysterols and cholestenoic acids, identified and quantified by LC-MS, in plasma of α-synuclein-injected mice.

		Concentration (ng/mL) ± SD								
**Identified structure after treatment with Cholesterol Oxidase**	**$[M]^+$ of GP *m/z* ± 5 ppm[a]**	**No injection (n=5)**	**Empty-vector (EV) injected (n=4)**	**α-synuclein injected ($1.7x10^{12}$ GC/mL) (n=5)**	**α-synuclein injected ($5.1x10^{12}$ GC/mL) (n=3)**	**Rt (mins)**	**P value (EV vs $1.7x10^{12}$ GC/mL α-synuclein)**	**P value (EV vs $5.1x10^{12}$ GC/mL α-synuclein)**	**Inferred structure prior to treatment with Cholesterol Oxidase**	**Inferred compound trivial name**
C-4-en-3-one	523.4419	179430.09 ± 17796.55	209166.09 ± 24606.55	200406.38 ± 14634.49	242815.17 ± 21618.58	11.36	0.559	0.116	C-5-en-3β-ol	Cholesterol
C-4,24-dien-3-one	521.4262	74.13 ± 25.37	246.46 ± 69.18	169.36 ± 69.98	198.85 ± 21.06	10.63	0.145	0.269	C-5,24-dien-3β-ol	Desmosterol
C-5-en-3β-ol-7-one[a]	534.4054	5.68 ± 1.48	4.53 ± 1.85	5.64 ± 0.64	5.49 ± 1.02	9.81	0.321	0.424	C-5-en-3β-ol-7-one	7-Oxocholesterol
C-4-en-3,24-dione[b]	537.4212	0.99 ± 0.21	0.85 ± 0.10	0.99 ± 0.23	1.03 ± 0.11	8.02			C-5-en-3β-ol-24S,25-epoxide	24-Oxocholesterol
C-4-en-24,25-diol-3-one[c]	555.4317	1.11 ± 0.34	1.21 ± 0.08	1.53 ± 0.77	1.51 ± 0.24	4.16			C-5-en-3β-ol-24,25-epoxide	24,25-Dihydroxycholesterol
		2.10 ± 0.47	2.06 ± 0.16	2.53 ± 0.99	2.54 ± 0.33		0.556 [d]	0.114 [d]	C-5-en-3β-ol-24S,25-epoxide	Total 24S,25-Epoxycholesterol[e]
C-4-en-24S-ol-3-one[f]	539.4368	5.73 ± 1.16	6.34 ± 0.56	6.39 ± 1.83	7.54 ± 0.37	18.28/19.34	0.957	0.019*	C-5-en-3β,24S-diol	24S-Hydroxycholesterol
C-4-en-25-ol-3-one[f]	539.4368	1.73 ± 0.40	1.98 ± 0.25	2.15 ± 0.51	1.91 ± 0.19	18.97	0.532	0.682	C-5-en-3β,25-diol	25-Hydroxycholesterol
C-4-en-26-ol-3-one[f]	539.4368	15.07 ± 4.68	13.00 ± 0.92	14.25 ± 3.68	13.21 ± 0.99	20.46	0.556 [d]	0.905 [d]	C-5-en-3β,(25R)26-diol	27-Hydroxycholesterol
C-4-en-7α-ol-3-one	539.4368	6.00 ± 2.64	6.86 ± 4.25	8.92 ± 6.41	5.88 ± 1.44	10.18/10.49	0.905 [d]	0.857 [d]	C-5-en-3β,7α-diol	7α-Hydroxycholesterol
C-4-en-6β-ol-3-one	539.4368	1.85 ± 1.16	1.73 ± 0.88	5.93 ± 8.77	1.94 ± 0.61	10.42	0.413 [d]	0.905 [d]	C-4-en-3β,6β-diol	6β-Hydroxycholesterol[g]
CA-4-en-3-one	553.4161	5.78 ± 1.68	5.31 ± 0.29	5.99 ± 1.06	4.20 ± 0.54	7.95	0.229	0.053	CA-5-en-3β-ol	3β-Hydroxycholestenoic acid
C-4-en-7α,25-diol-3-one[a]	550.4003	0.07 ± 0.02	0.08 ± 0.01	0.09 ± 0.03	0.11 ± 0.02	5.96/ 6.74	0.407	0.195	C-5-en-7α,25-diol-3-one	7α,25-Dihydroxycholestenone
C-4-en-7α,25-diol-3-one	555.4317	0.09 ± 0.02	0.09 ± 0.02	0.10 ± 0.04	0.09 ± 0.01	5.99/6.77	>0.999 [d]	>0.999 [d]	C-5-en-3β,7α,25-triol	7α,25-Dihydroxycholesterol
C-4-en-7α,26-diol-3-one[a]	550.4003	0.20 ± 0.08	0.14 ± 0.03	0.18 ± 0.09	0.19 ± 0.10	6.50/ 7.32	0.905 [d]	0.857 [d]	C-5-en-7α,(25R)26-diol-3-one	7α,(25R)26-Dihydroxycholestenone
C-4-en-7α,26-diol-3-one	555.4317	0.01 ± 0.01	0.01 ± 0.01	0.02 ± 0.01	0.02 ± 0.02	6.46/ 7.31	0.310 [d]	0.457 [d]	C-5-en-3β,7α,(25R)26-triol	7α,(25R)26-Dihydroxycholesterol
CA-4-en-7α-ol-3-one[a]	564.3796	53.59 ± 18.81	39.78 ± 6.660	55.78 ± 17.20	44.00 ± 16.90	6.20/ 6.98	0.110	0.715	CA-4-en-7α-ol-3-one	7α-Hydroxy-3-oxocholestenoic acid
CA-4-en-7α-ol-3-one	569.4110	1.47 ± 2.01	0.26 ± 0.52	2.35 ± 3.05	0.23 ± 0.41	6.20/ 6.98	0.365 [d]	>0.999 [d]	CA-5-en-3β,7α-diol	3β,7α-Dihydroxycholestenoic acid

Footnotes to the table:

Systematic nomenclature adopted according to LipidMaps http://www.lipidmaps.org/. Many oxysterols when derivatised give twin peaks, where appropriate both retention times are given.

C = cholestene, a number preceding "en" indicates the location of carbon-carbon double bond(s), a number(s) preceding "ol (diol, etc)" or "one" indicates the location of hydroxy and oxo groups, respectively. CA = cholestenoic acid.

ng/mL = nanograms per millilitre; SD = standard deviation; NS = no surgery; EV = empty vector; GC/mL = genome copies per millilitre; Rt = retention time.

[a] - Where compounds have an odd numbered integer *m/z* they have been derivatised with [$^2H_5$]GP following cholesterol oxidase treatment. Where the integer *m/z* is an even number, the compounds have been derivatised with [$^2H_0$]GP in the absence of cholesterol oxidase treatment.

[b] - Isomerisation product of 24S,25-epoxycholesterol formed during derivatisation.

[c] - Hydrolysis product of 24S,25-epoxycholesterol formed during derivatisation.

[d] – Mann-Whitney U test used to test data due to the data not being normally distributed (Shapiro-Wilk normality test). All other data sets showed normal distribution of data, and therefore were tested with an unpaired t test with Welch's correction.

[e] - Total 24S,25-epoxycholesterol is the sum of 24-oxocholesterol, 24,25-dihydroxycholesterol and any residual 24S,25-epoxycholesterol.

[f] - Quantified using 37-minute LC gradient, rather than the regular 17-minute gradient.

[g] - 6β-Hydroxycholesterol is a dehydration product of cholestane-3β,5α,6β-triol. The triol can be formed from 5,6-epoxycholesterol during the derivatisation process or could be endogenously formed non-enzymatically.

Table S2. Levels of 24-Oxocholesterol, 24,25-Dihydroxycholesterol and total 24*S*,25-epoxycholesterol, identified and quantified by LC-MS, in plasma of patients with hereditary spastic paraplegia type 5 (SPG5).

		**Concentration (ng/mL) ± SD**				
**Identified structure after treatment with Cholesterol Oxidase**	**$[M]^+$ of $[^2H_5]$GP *m/z* ± 5 ppm**	**SPG5 patients (n=8)**	**NIST SRM 1950**	**Rt (mins)**	**Inferred structure prior to treatment with Cholesterol Oxidase**	**Inferred compound trivial name**
C-4-en-3,24-dione[a]	537.4212	0.38 ± 0.07	0.53	7.82	C-5-en-3β-ol-24S,25-epoxide	24-Oxocholesterol
C-4-en-24S,25-diol-3-one[b]	555.4317	5.48 ± 0.99	0.17	5.14	C-5-en-3β-ol-24S,25-epoxide	24,25-Dihydroxycholesterol
		**5.86 ± 1.03**	**0.70**		**C-5-en-3β-ol-24S,25-epoxide**	**Total 24S,25-epoxycholesterol[c]**

Footnotes to the table:

Systematic nomenclature adopted according to LipidMaps http://www.lipidmaps.org/.

C = cholestene, a number preceding "en" indicates the location of carbon-carbon double bond(s), a number(s) preceding "ol (diol, etc)" or "one" indicates the location of hydroxy and oxo groups, respectively.

ng/mL = nanograms per millilitre; SD = standard deviation; Rt = retention time.

NIST SRM – National Institute of Standards and Technology (NIST) standard reference material (SRM)

[a] - 24S,25-Epoxycholesterol isomerises to 24-oxocholesterol during the derivatisation process.

[b] - 24,25-Dihydroxycholesterol is a hydrolysis product of 24S,25-epoxycholesterol.

[c] – Total 24S,25-epoxycholesterol is the sum of 24-oxocholesterol, 24,25-dihydroxycholesterol and any residual 24S,25-epoxycholesterol.
